# Early Diagnosis of Oral Cancer: A Complex Polyhedral Problem with a Difficult Solution

**DOI:** 10.3390/cancers15133270

**Published:** 2023-06-21

**Authors:** Isabel González-Ruiz, Pablo Ramos-García, Isabel Ruiz-Ávila, Miguel Ángel González-Moles

**Affiliations:** 1 School of Dentistry, University of Granada, 18071 Granada, Spain; isagonzru@gmail.com; 2 Instituto de Investigación Biosanitaria ibs.GRANADA, 18012 Granada, Spain; iruizavila@gmail.com; 3 Hospital Universitario San Juan de Reus, CAP Marià Fortuny, 43204 Tarragona, Spain

**Keywords:** oral cancer, early diagnosis, diagnostic delay, prognosis

## Abstract

**Simple Summary:**

Oral and oropharyngeal cancers account for a worldwide incidence of 377,713 and 98,412 new cases annually and 177,757 and 48,143 deaths per year, respectively. Approximately 90% of oral malignancies are squamous cell carcinomas, showing a 5-year mortality rate still close to 50%. The poor prognosis of oral cancer is mainly related to its late diagnosis in advanced stages (stage III/IV), in which treatment is not effective. Therefore, reducing the delay in the diagnosis of oral cancer is an essential step in order to improve the outcomes and outlooks of patients affected by this disease. In this article, the diagnostic delay of oral patients is critically reviewed, jointly with their main reasons, difficulties, and future strategies for improvement.

**Abstract:**

Oral and oropharyngeal cancers are a growing problem, accounting for 377,713 and 98,412 new cases per year all over the world and 177,757 and 48,143 deaths annually, respectively. Despite the substantial improvement in diagnostic procedures and treatment techniques in recent years, the mortality rate has not decreased substantially in the last 40 years, which is still close to 50% of cases. The major cause responsible for this high mortality is associated with the high percentage of oral cancers diagnosed in advanced stages (stages III and IV) where the treatment harbors poor efficacy, resulting in challenges, mutilations, or disability. The main reason for cancer to be diagnosed at an advanced stage is a diagnostic delay, so it is critical to reduce this delay in order to improve the prognosis of patients suffering from oral cancer. The causes of oral cancer diagnostic delay are complex and concern patients, healthcare professionals, and healthcare services. In this manuscript, oral cancer diagnostic delay is critically reviewed based on current evidence, as well as their major causes, main problems, and potential improvement strategies.

## 1. Introduction

Oral and oropharyngeal carcinomas represent a major problem in head and neck pathology due to their frequency as well as the serious consequences that still result from their involvement. Data derived from the most relevant organizations and institutions show that these neoplasms represent 377,713 and 98,412 new cases worldwide per year and 177,757 and 48,143 deaths per year, respectively (GLOBOCAN, IARC, WHO) [1]. It is very relevant and, in some sense, difficult to assume and explain the high mortality rate of these tumors, despite the substantial improvement in diagnostic procedures and treatment techniques in recent years. The mortality rate is still close to 50% of cases, jointly considering all carcinomas in these locations. It is also important to keep in mind that this mortality increases notably if any of the known factors that worsen the prognosis appear, i.e., the involvement of cervical lymph nodes. Neoplasms affecting the oral cavity and oropharynx belong to the group of squamous cell carcinomas (OSCC), which are basically related to tobacco and alcohol consumption and, in the case of the oropharynx, also to the infection by the oncogenic human papillomavirus (especially types 16 and 18) [2,3]. Another remarkable aspect, hard to clear up, concerns the high percentage of patients, close to 50%, who are diagnosed in advanced stages (T3 and T4, 50%; N+, 47%) in which survival declines drastically, and the applicable therapies are generally aggressive and mutilating, notably conditioning the quality of life of these patients [4,5]. This fact is difficult to understand if we take into account the anatomical location of this carcinoma, an easily explorable area that should be routinely examined by a multitude of specialists, including dentists, family physicians, dermatologists, otolaryngologists, maxillofacial surgeons, etc. Evidence points to the fact that the cause of the slow improvement in the prognosis of oral and oropharyngeal cancer that we have been facing for many years is essentially due to the delay in the diagnosis of these tumors. The results of systematic reviews and primary-level studies carried out with good methodological quality [6,7,8,9,10,11,12,13] indicate that this delay results in the diagnosis of carcinomas in more advanced stages, and some case series also relate this fact to higher mortality, there being broad agreement, as some studies report [7,14], on the influence of early diagnosis and treatment in improving the survival of these patients. Therefore, a key objective in the management of this pathology should be to achieve early diagnosis in most of these patients. We should ask ourselves why we are failing in this objective, both in developing countries and in developed countries of the first world. The reasons for this fact are probably multiple and derive from the fact that there are also many different scenarios and actors involved that are responsible for the late diagnosis of these tumors.

This review presents the probable causes that condition the late diagnosis of oral cancer and some strategies that could improve it, as well as a forecast of what is expected for the coming years, all based on published evidence-based studies and case series conducted with good methodological quality, under the personal vision of a clinical and research group, and multidisciplinary (family physicians, dentists, pathologists, and specialists in oral medicine), with experience in this field.

## 2. When Should We Consider That an Oral Carcinoma Has Been Early Diagnosed?

**Figure 1:**
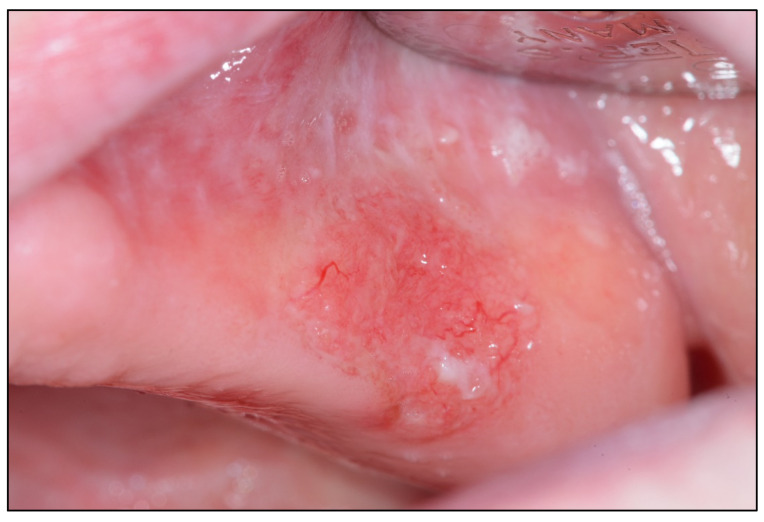
Oral cavity tumor <2 cm in greatest diameter (T1c).

**Figure 2:**
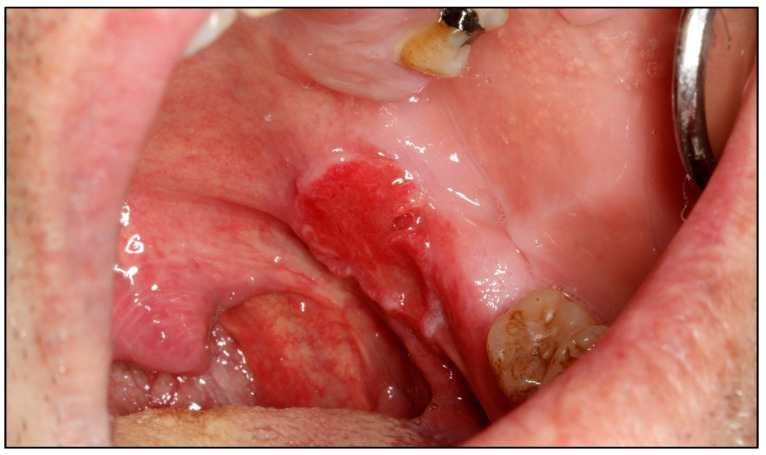
Tumor of approximately 2 cm in diameter, located in the retromolar trigone and tonsillar pillar, an area rich in lymphatic vessels.

**Figure 3:**
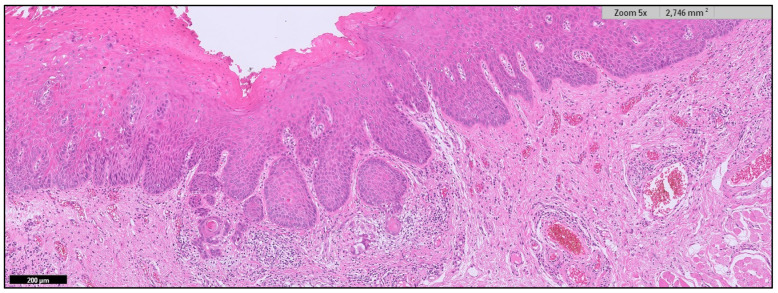
Oral carcinoma with less than 5 mm depth of invasion (T1).

Logic dictates that the concept of incipient carcinoma should be linked to its size. Thus, it seems reasonable to assume that incipient carcinomas should be small tumors. However, this could have exceptions linked to the speed of tumor growth, e.g., proliferative capacity; a slow-growing tumor could remain small in size for prolonged periods of time and vice versa. However, if we accept as a general rule that an incipient carcinoma is small, then what size should an oral carcinoma be to consider an early diagnosis has been made? T1 tumors in the AJCC classification are those that measure ≤ 2 cm in their greatest diameter both at the time of patient examination (T1c) and at the measurement of the operative specimen (T1p) [15,16] (Figure 1). Is it known that these carcinomas have a better prognosis than larger carcinomas [17]? However, tumors 2 cm in diameter larger than the one shown in the image (Figure 2), especially in certain locations, including the tongue, the floor of the mouth, and tonsillar pillars, will probably have a less favorable prognosis despite being classified as T1. The reason why large tumors have a worse prognosis is related to their greater capacity to metastasize, especially in the lymph nodes of the neck, and this depends on their capacity to infiltrate the tissue in depth and to invade the blood vessels and especially the lymphatic vessels. Our research group has reported in tongue cancer that a depth of invasion greater than 3 mm is associated with significantly higher mortality. Finally, because of the above, the AJCC in its latest TNM classification [15,16] has considered the depth of invasion as a key parameter to measure tumor size, requiring, in addition to a size less than 2 cm, a depth of invasion ≤ 5 mm to consider a tumor as T1 (Figure 3). Therefore, from our point of view, an oral carcinoma should be considered to have been diagnosed early when it measures less than 2 cm and presents ≤ 5 mm depth of invasion.

In spite of the above, the concept of diagnostic delay has often been linked to the time parameter, i.e., a diagnosis of oral cancer would have been late if too much time has elapsed between the first symptoms of the disease and its definitive diagnosis [8,14,18,19,20,21,22,23,24]. Obviously, this raises important problems, the first of which relates to the definition of a time cut-off point from which a cancer diagnosis would be considered to have been delayed, and, in general, the choice of that time point has been arbitrary and without sufficient scientific basis (more than 30 days for example) [25,26,27,28,29,30]. Although the passage of time increases the probability that a tumor will present a worse prognosis, what really conditions the prognosis of the tumor is its aggressiveness, in other words, its capacity to spread to adjacent tissues, infiltrating and destroying them, and to affect lymphatic vessels to metastasize to neck nodes, which is associated with its depth of invasion. Thus, in a parallel and complementary manner to the concept of incipient carcinoma, the concept of late diagnosis should be considered; in our opinion, the diagnosis is late if the tumor has more than a 5 mm depth of invasion.

## 3. Diagnosis of Oral Cancer: A Tortuous Path

The process of diagnosis of oral cancer involves the development of a series of clinical events affecting the oral mucosa, which are initially subtle but progressively become more evident. These events are perceived by the patient who will have to transfer them to a healthcare structural framework, which will finally conclude in the diagnosis of cancer. Undoubtedly, this is a tortuous path involving multiple actors that can develop in very diverse sociocultural and healthcare scenarios and is affected by different conditioning and modifying factors. It is clear that the study of the reasons that delay diagnosis, that is, slow down the progression of events that must occur to reach a definitive diagnosis of oral cancer, is complex. The Aarhus Declaration [31], published in 2012, is a consensus document on the different events that unfold on the path to oral cancer diagnosis, attending to its actors, its key periods and moments, and its conditioning factors. This consensus document provides very useful information to understand, analyze, and fight against the delay in the diagnosis of oral cancer. Patients should detect the changes that are occurring in their oral mucosa, consider them abnormal, accept that they need professional help, and arrange a first visit with a health professional, usually a family physician or a dentist. Once the patient is received for the first time by a health professional, the diagnostic interval begins [31], which includes the first consultation, the referral to a specialist (frequently a maxillofacial surgeon, an oral medicine specialist, or an otolaryngologist), the first consultation with the specialist and the establishment of a definitive histopathological diagnosis. Finally, the patient should undergo the most appropriate treatment for the tumor, which should be planned by an oncological committee. As we will see, each of these intervals can be a cause of diagnostic delay. Finally, there are conditioning factors that could also contribute to a delay in diagnosis; these depend on the tumor itself, for example, its location in areas difficult to explore, like the posterior edge of the tongue, or on the patient, such as poverty, lack of education, etc. [10,28,32,33,34,35].

## 4. Why Is Oral Cancer Diagnosis So Often Delayed? How to Fight It?

As we have already mentioned, oral cavity cancer is diagnosed with a very high frequency (approximately 50% of cases) at very large sizes (T3 or T4) or in stages in which the lymph nodes of the neck are affected (N+). In these patients, the prognosis is markedly poorer, with the probability of death being very high and the consequences of treatment being very negative for the patient’s quality of life. These figures are unacceptable and, to a large extent, inexplicable, especially if we take into consideration that they have not changed substantially in the last 50 years; this leads us to think that the delay in the diagnosis of oral cancer is probably due to reasons that are difficult to modify.

**Figure 4:**
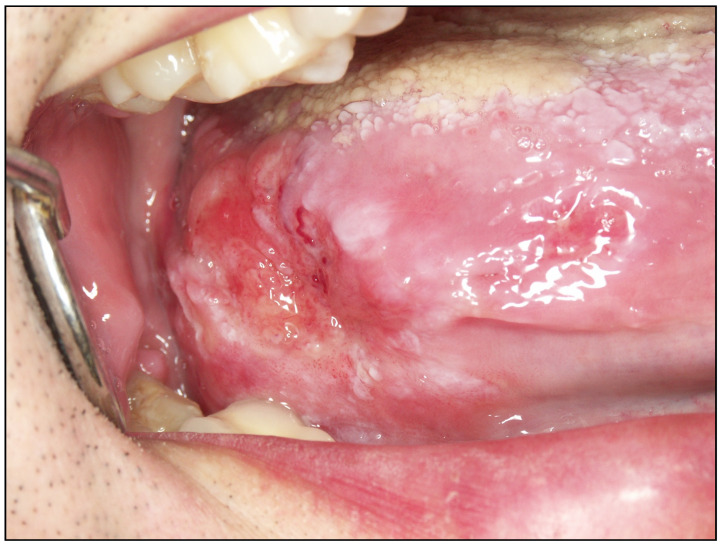
This carcinoma of the lateral and posterior border of the tongue grew silently to a large size at the time of diagnosis.

**Figure 5:**
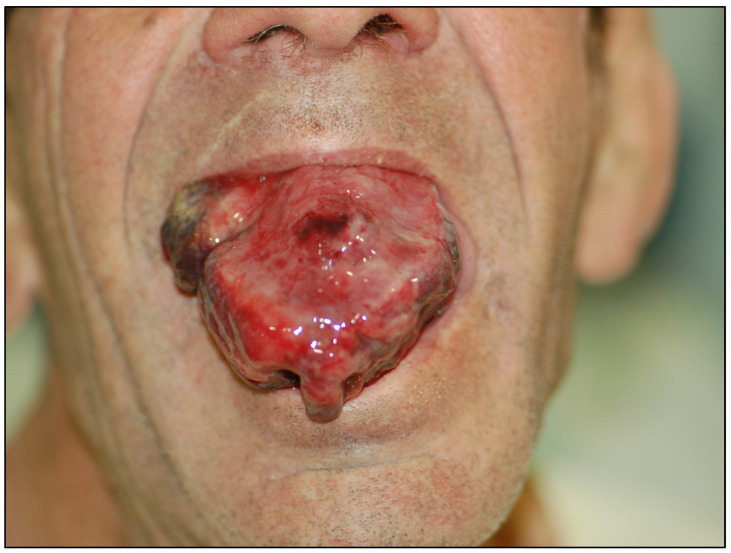
Oral cavity cancer of a monstrous size in a patient of low intellectual level, of pastor profession who lived in an isolated situation.

*The causes of diagnostic delay may depend on the patient*. The time period in which the diagnosis of oral cancer depends solely on the patient is often unacceptably long. It has been reported that, on average, patients spend 104.9 days from the time they perceive the first signs or symptoms of the tumor until they seek help [36]. It should also be taken into account that this period is probably longer as a consequence of conditioning factors derived from the tumor; many carcinomas in their early stages are asymptomatic and are located in areas that are difficult for the patient to detect (Figure 4). It is common for cancer patients to initially attribute their symptoms or lesions to trivial causes (friction, infections, trivial ulcers, etc.) [22,37]; there is then a delay in perceiving the signs and symptoms as something abnormal. Fear of receiving the diagnosis of cancer, of suffering the consequences of treatment, or of facing the possibility of dying is also frequently in the background of this delay. On the other hand, the patient’s own social and cultural circumstances may delay diagnosis at these stages. In particular, the following conditions may delay diagnosis: poverty and belonging to depressed social strata; senescence in those who live alone, a fact extremely frequent in our society, to such an extent that some advanced countries are creating ministries to combat isolation; senescence in patients living in institutions with a low level of care; homeless people; immigrants, especially illegal ones; refugees due to war or climate-related conflicts; and people with cognitive impairment may have serious problems in interpreting the initial symptoms of cancer as abnormal and/or in seeking help [14,38,39,40,41,42] (Figure 5). A reflection on the causes of patient-dependent diagnostic delay will lead the reader to the conclusion that its correction and the shortening of this interval is very difficult and will often be unsuccessful. How do we fight against poverty, social exclusion, or the other causes that have been mentioned? An effort by governments is needed to better inform the population about the importance of oral cancer and to improve the living conditions of citizens.

**Figure 6:**
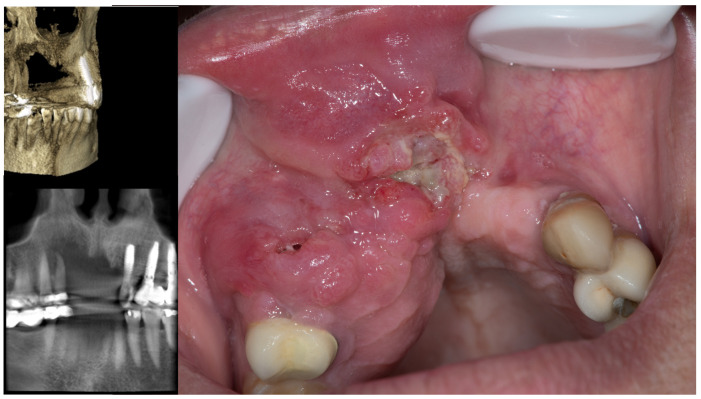
This patient with a monstrous oral carcinoma was treated for months by a dentist under the diagnosis of the inflammatory and infectious process, applying antibiotics and performing teeth extractions, with the aim of placing dental implants in the area. Only indifference and an unacceptable lack of commitment to the health of patients can explain these cases.

*The causes of diagnostic delay in oral cancer may be related to healthcare providers and deficiencies in public health services.* Healthcare providers, especially those on the front line in the process of diagnosing oral cancer, such as family physicians and dentists, may be responsible for diagnostic delays for a variety of reasons, including the following: a frequent manifest ignorance of the signs and symptoms presenting with early-stage oral carcinomas and oral lesions at risk of developing cancer, i.e., oral potentially malignant disorders (OPMDs) [43,44], and a frequent unacceptable lack of awareness, and even neglect, of the possibility that a patient may have an oral carcinoma [45] (Figure 6). It is also common for some healthcare providers to prescribe unjustified treatments for the clinical picture, essentially based on a variety of mouthwashes, e.g., corticosteroids, chlorhexidine, hyaluronic acid, or antibiotics, or even to make a new appointment after some time without taking any specific action in the hope of some improvement (“let us wait and see how your lesion evolves”), which constitutes a negligent attitude only justified in their ignorance and indifference. Primary care physicians could focus more on the chronic pathologies that these patients frequently present, many of them related to tobacco consumption, which is also the essential cause of oral cancer (pulmonary diseases, arterial hypertension, cardiovascular pathologies, etc.), neglecting the thorough examination of the patient [21]. Finally, it is common that very few biopsies are performed, both in primary care and by dentists in the public system or in private practice, which also extends the time of diagnosis; only between 7% and 32% of dentists in different countries perform oral biopsies [46,47,48,49,50,51,52].

The main problem in public health services, which has a determining influence on the delay in the diagnosis of oral cancer, is the saturation and work overload of healthcare providers. This hinders the normal performance of their functions, which include taking a correct clinical history and conducting a meticulous examination of the oral mucosa, which should be carried out on all patients who come to a health service, whatever the reason for their consultation. This saturation also affects later periods of the diagnostic process, such as, for example, attention by specialists to confirm the presence of suspected lesions and biopsy them, or during the process of definitive diagnosis in a pathology service.

*The knowledge that clinicians should have in order to improve the rates of early diagnosis of oral cancer.* In 2020, an international group of clinicians and researchers with expertise in oral cancer and oral precancer, convened by the WHO’s collaborative center for the study of oral cancer, met in Glasgow with the mission to update the concepts of oral lesions that are predisposed to the development of cancer, called oral potentially malignant disorders (OPMD), and to compile and present to the scientific community the factors that increase the risk of their progression to cancer [53]. Table 1 presents the oral lesions that should be considered today as OPMD. The clinical presentation of OPMD was discussed in Appendix A.

*Strategies for improvement in the early diagnosis of oral cancer.* Strategies to improve the early diagnosis of oral cancer should essentially aim to find cases of cancer at stages where no symptoms are yet present or at the earliest stages of cancer development where symptomatology is minimal.

*Active search for cases* defines the clinical procedure by which oral carcinomas are diagnosed that have generated some symptoms that, having been interpreted as abnormal by the patient, awaken in them the need to seek help [54]. Improving the performance of this early diagnosis strategy implies, as we have mentioned, improving the sociocultural level of patients, increasing information to the population on the importance of early diagnosis of oral cancer, increasing the training of family physicians and dentists, and encouraging them to fulfill their obligation in this regard.

Oral cancer screening programs are public health organized programs that, under precise indications, have great potential to improve health outcomes related to a specific problem. They are based on non-diagnostic screening tests that aim to detect abnormalities that justify and accelerate the referral of a patient for a definitive diagnosis. The essential screening test in oral cancer early detection programs is visual inspection with palpation of the oral mucosa and neck [54]. These include *opportunistic screening programs*, which are not systematic, and have as their target population those patients who come to the office for other health reasons. These patients should undergo a visual examination of the oral cavity. There is insufficient evidence on the efficacy of these programs, although the results of some studies on the subject seem to support them [55,56]. *Population screening programs* are systematic for the entire preselected target population, regulated and protocolized, and subject to continuous evaluation in a framework dependent on public health systems. There is insufficient evidence to indicate these programs in oral cancer [57,58].

The main problems of oral cancer screening programs concern the high rate of false-positive results, which generates unnecessary public expenditure and stress for the patient; their ineffectiveness in countries with a low incidence of oral cancer, the low adherence of patients to referrals to specialists for confirmatory diagnosis [59,60,61,62,63]; the heterogeneity in the training of examiners; and the low level of resources in countries with higher incidences of oral cancer, which would be the main beneficiaries of this type of program.

## 5. Final Conclusions and Future Outlook

A reflection on the fundamental causes related to the late diagnosis of most oral cavity carcinomas leads to the conclusion that it is difficult to solve this serious problem, which significantly affects the prognosis of these patients and their quality of life. *How do we fight against patient-dependent reasons for diagnostic delay?* A general improvement in the sociocultural status of the world’s population, which has a determining influence on the interpretation of the first symptoms of cancer, is a utopia that will probably never be overcome. Getting a patient with a lesion suspected to be cancer to overcome the fear of receiving the news and its consequences is a very difficult task that depends considerably on the personality of each patient. Only widespread public information programs on the importance of oral cancer or the consequences of delayed diagnosis can alleviate this aspect. *How do we fight against the causes of delayed diagnosis that depend on healthcare providers and health services?* Many of these reasons concern the lack of knowledge of the health professionals who are on the front line of care for these patients (family physicians and dentists) about the initial signs and symptoms of oral cancer. This aspect would improve if more emphasis was placed on these aspects in medical and dental degrees and if the implementation of continuing education programs on oral cavity cancer was encouraged by the public bodies and professional associations that lead with healthcare. However, part of the responsibility for this aspect derives from the indifference and lack of commitment of many health professionals to the early diagnosis of oral cancer; this is obviously very difficult to correct, and only penalizing legislation in this regard could be efficient to some extent. It should also be pointed out that primary care physicians in particular are unacceptably overworked, which has a very negative impact on their ability to perform their duties with dignity. This fact can only be solved by an effort on the part of governments to allocate public resources to healthcare and by a judicious organization of the activities of healthcare providers. 

Achieving an early diagnosis of oral cancer is also significantly challenged by the inefficacy of mass population-based screening programs for this type of tumor, which is not very prevalent in many countries of the world, with opportunistic detection being the type of screening that has proven to be most effective. Improving this type of screening concerns all actors involved in achieving an early diagnosis of oral cancer. It would be optimal if governments improved their healthcare programs to make it easier for professionals to have the training, time, and healthcare resources to perform this opportunistic screening on a widespread basis for all patients presenting for any reason. Healthcare providers should be made aware of the importance of the problem and show a proactive and diligent attitude toward the early diagnosis of cancer. Finally, a crucial point in this matter falls on the patients who should become aware of the importance of this aspect of their health and attend information programs, modify their risk habits and follow the recommendations of healthcare providers.

## Figures and Tables

**Table 1:** Malignant transformation of oral potentially malignant disorders reported in the systematic reviews and meta-analyses published in the Special Issue organized by the World Health Organization Collaborating Centre for Oral Cancer.

Oral Potentially Malignant Disorders	Sample Size (Primary-Level Studies)	Number of Patients	Malignant Transformation *
Oral Leukoplakia	n = 24 **	16,192	PP = 9.8% (95% CI: 7.9–11.7)
Oral Lichen Planus	n = 10 ***	3206	PP = 2.28% (95% CI = 1.49–3.20)
Oral Lichenoid Lesions	n = 3	197	PP = 2.11% (95% CI = 0.01–6.33)
Proliferative Verrucous Leukoplakia	n = 17	474	PP = 43.87% (95% CI = 31.93–56.13)
Oral Submucous Fibrosis	n = 9	6337	PP = 4.2% (95% CI: 2.7%–5.6%)

* This table only integrates those OPDMs for which there is scientific evidence of their malignant transformation proportions studied through meta-analyses and published in the WHO Collaborating Centre for Oral Cancer Special Issue. ** Published in the last 5 years. *** Based on 10 highest quality studies selected out of 89 publications. Abbreviations: WHO, World Health Organization; PP, pooled proportions; CI, confidence intervals.

**Figure A1:**
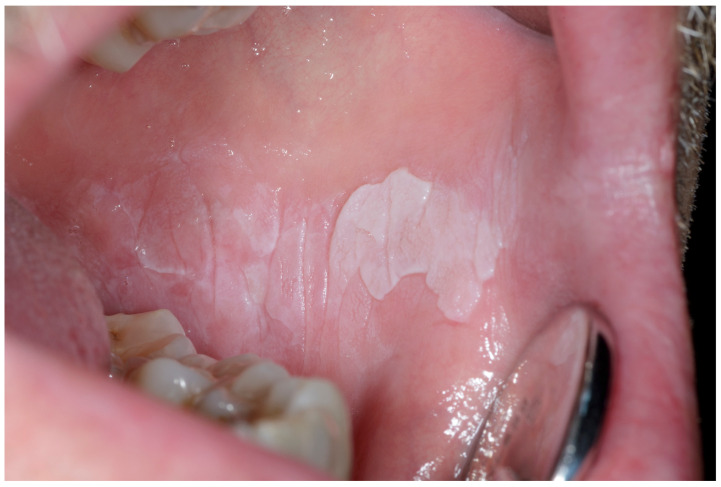
Homogeneous leukoplakia of the oral mucosa.

**Figure A2:**
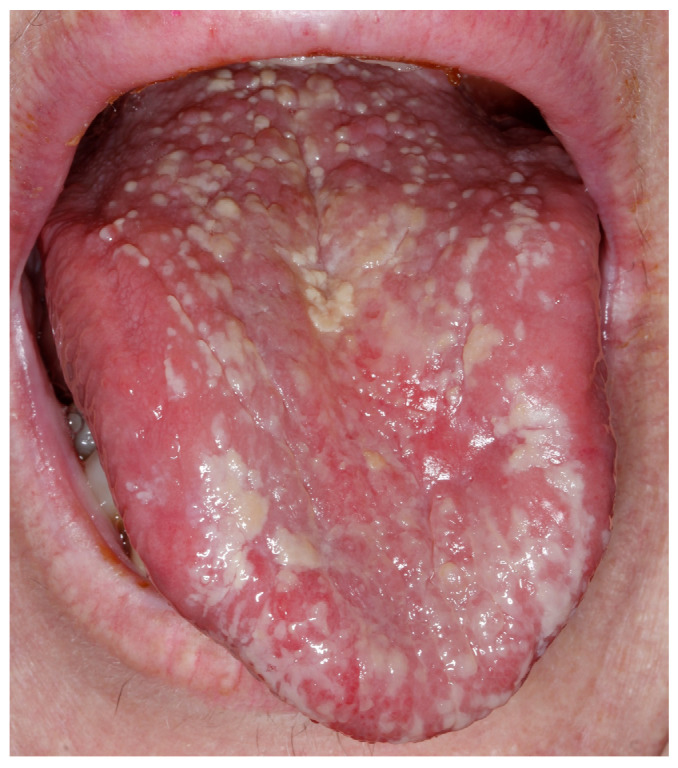
Pseudomembranous candidiasis.

**Figure A3:**
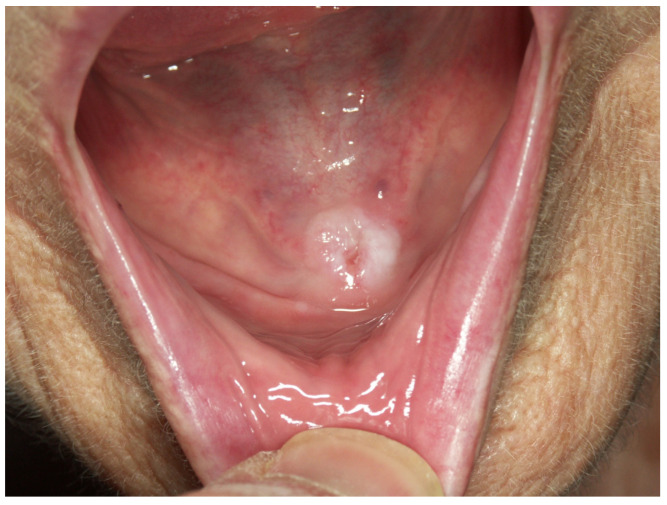
Frictional lesion on the floor of the mouth due to the friction of an ill-fitting prosthesis.

**Figure A4:**
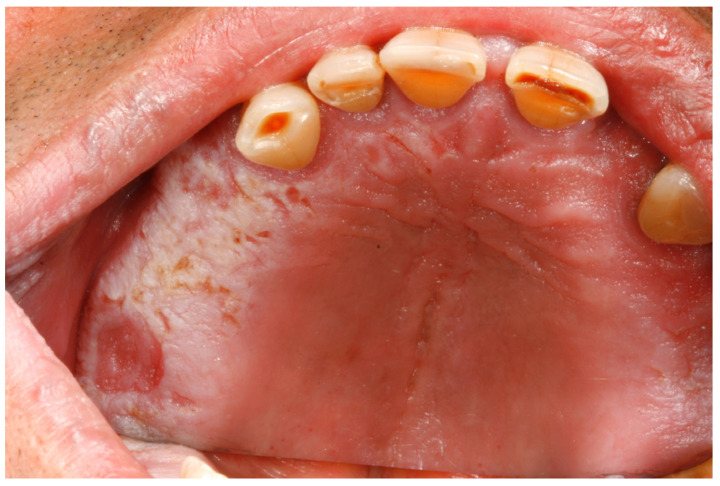
Frictional lesion on the alveolar ridge due to chewing with edentulous gingiva.

**Figure A5:**
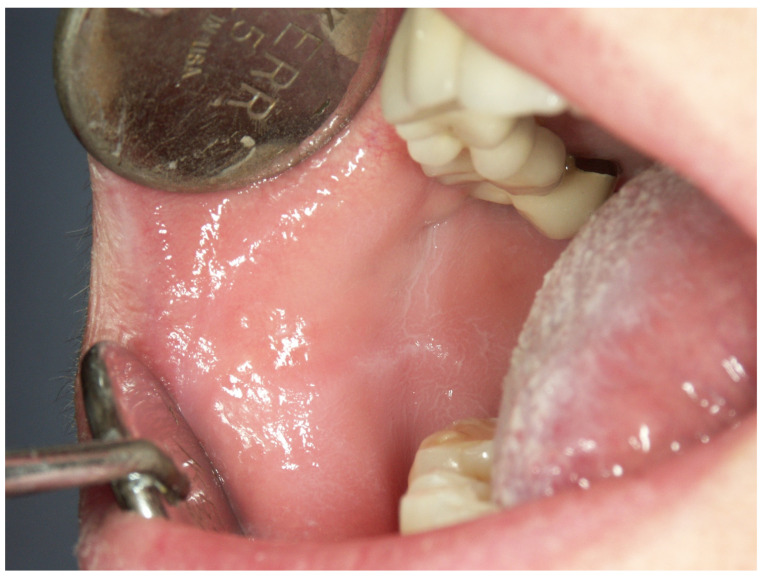
Leukoedema of the oral mucosa.

**Figure A6:**
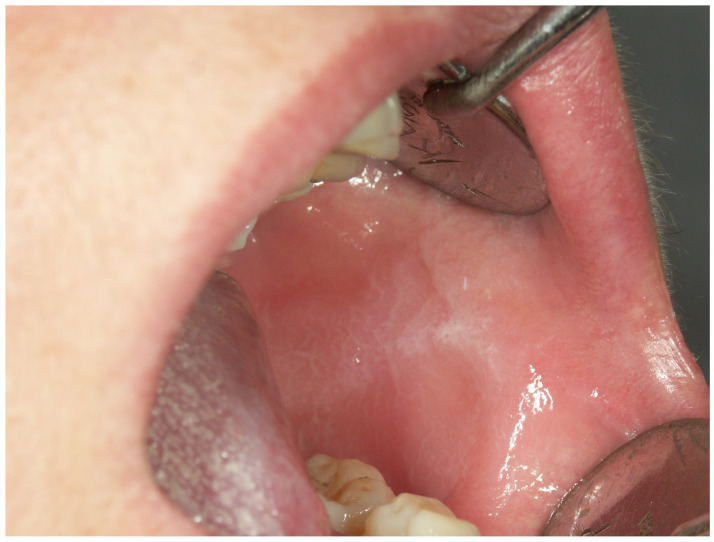
Alba line.

**Figure A7:**
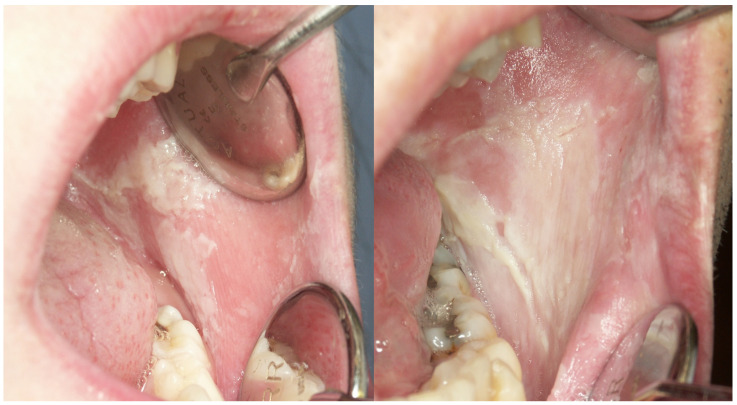
White spongy nevus in an 11-year-old girl (**left**) and in her father (**right**).

**Figure A8:**
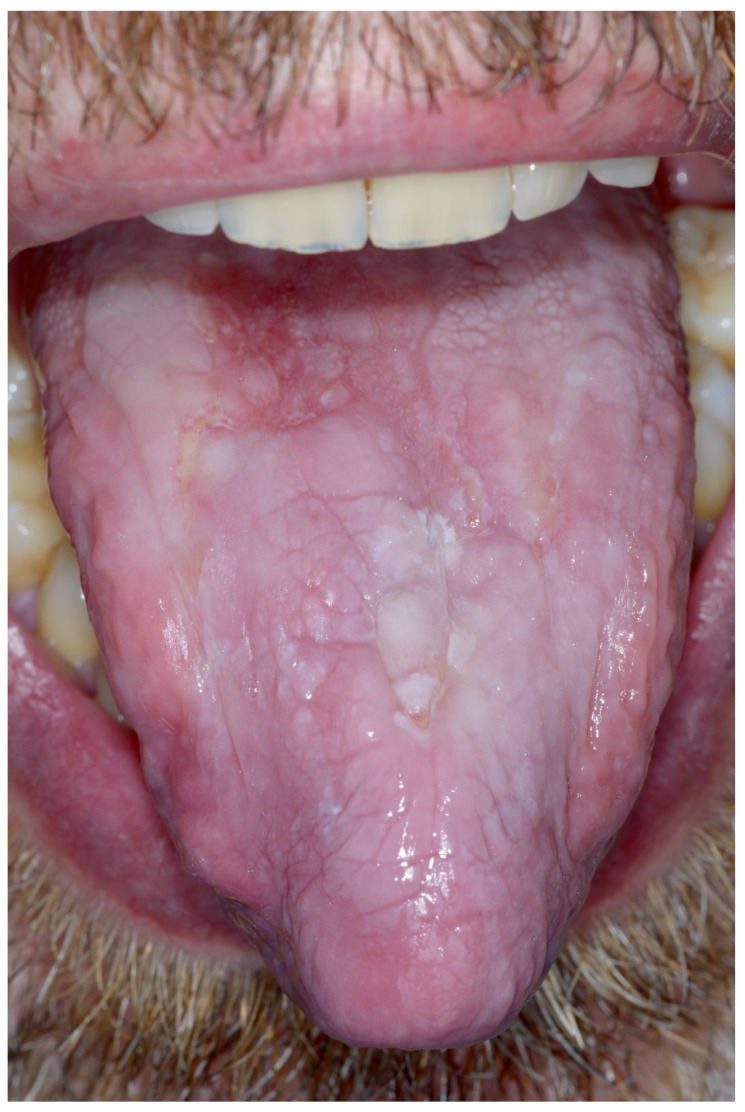
Leukoplakic images in a patient with dyskeratosis congenita.

**Figure A9:**
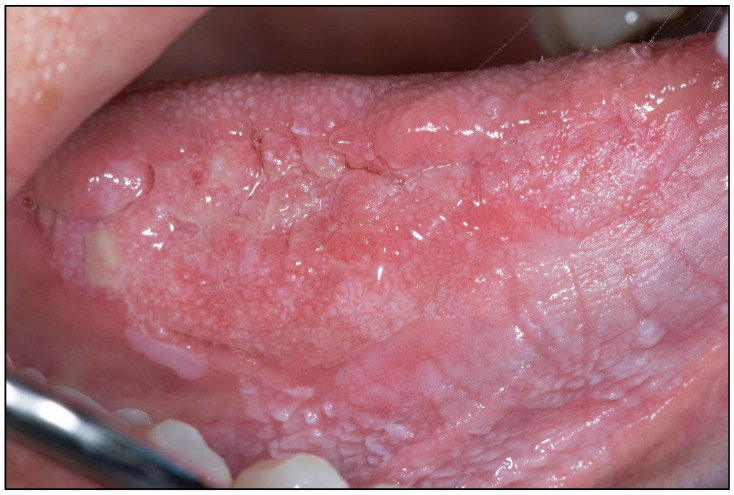
Oral carcinoma with granular appearance in a patient with oral leukoplakia.

**Figure A10:**
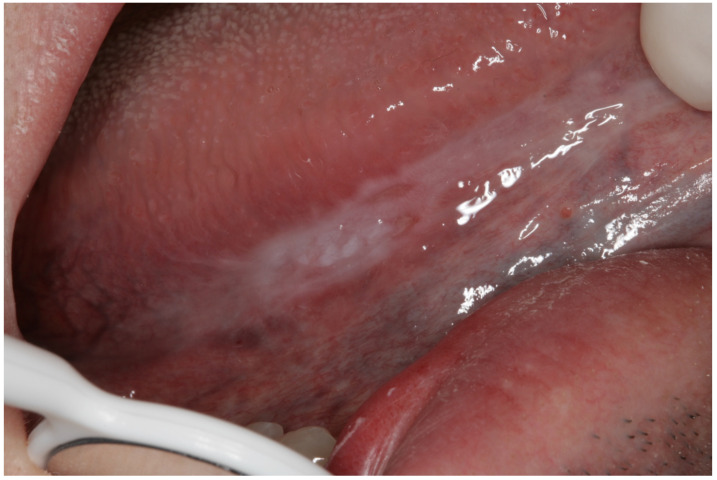
Incipient oral carcinoma with leukoplakia appearance.

**Figure A11:**
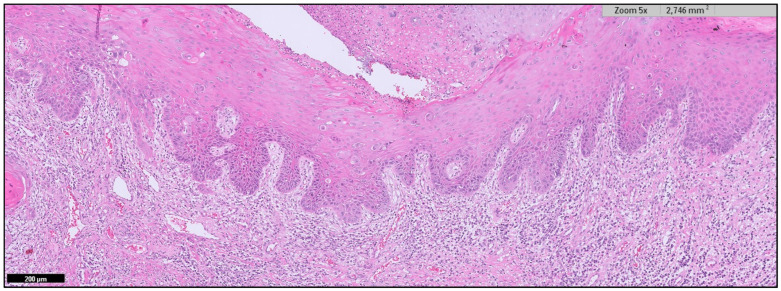
Images of severe dysplasia/carcinoma in situ.

**Figure A12:**
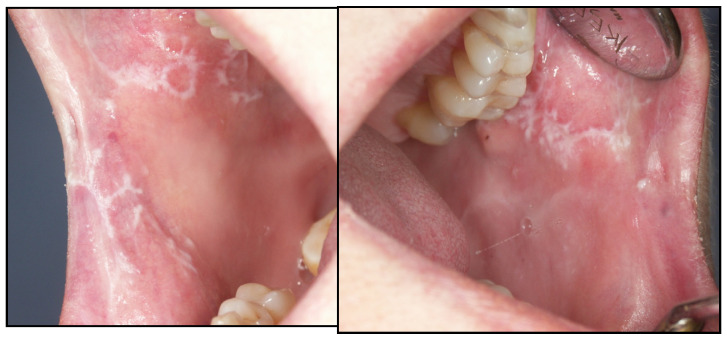
Bilateral reticular OLP.

**Figure A13:**
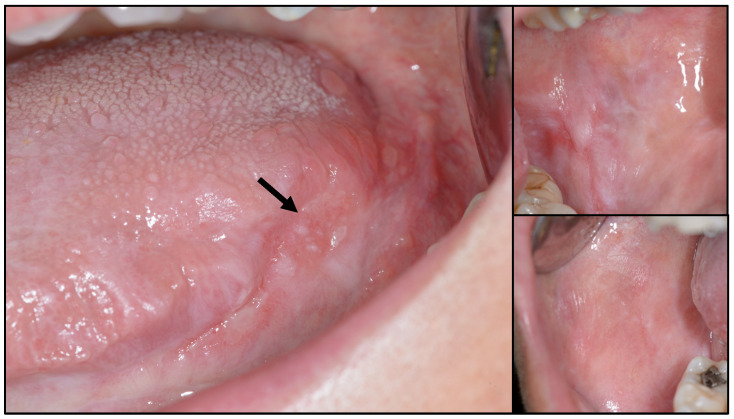
Incipient carcinoma developed in a patient with OLP. It presents granular appearance.

**Figure A14:**
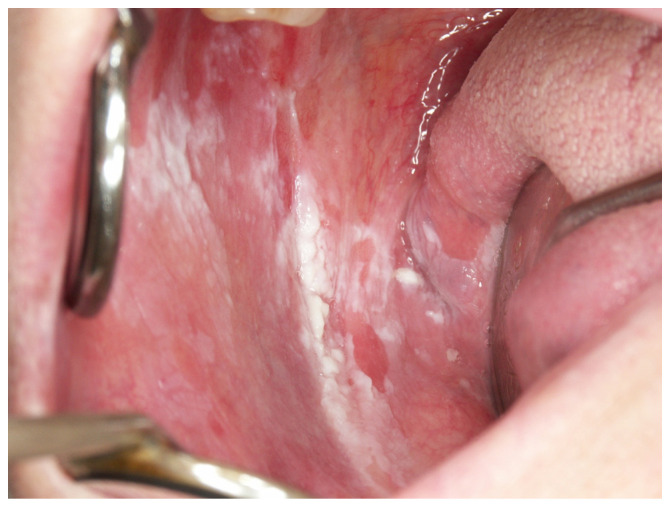
Image of PVL that externally affects the right buccal mucosa and other areas of the oral mucosa. There are white areas, in some areas thickened and some reddened areas.

**Figure A15:**
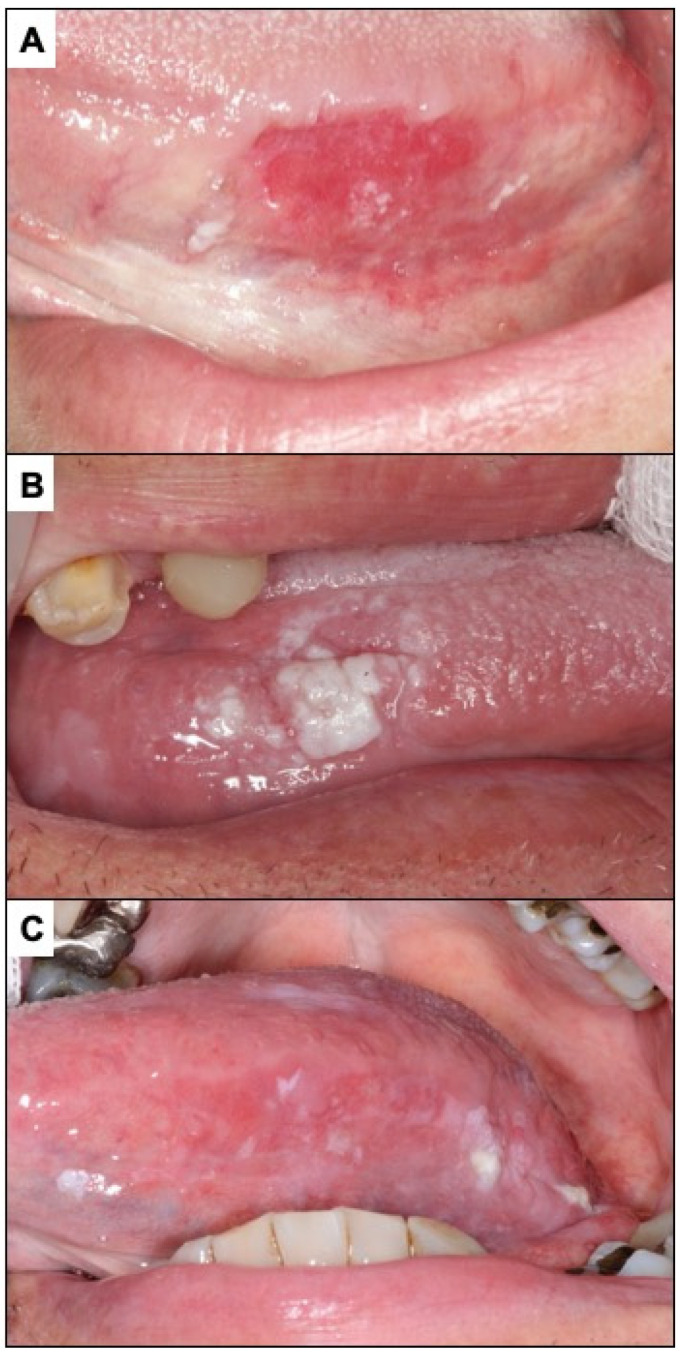
(**A**) Erythroplasia of the lateral border of the tongue. (**B**) Oral carcinoma with leukoplakia-like appearance on the lateral border of the tongue. (**C**) Oral carcinoma with a red and white appearance. Reproduced with permission from [67].

**Figure A16:**
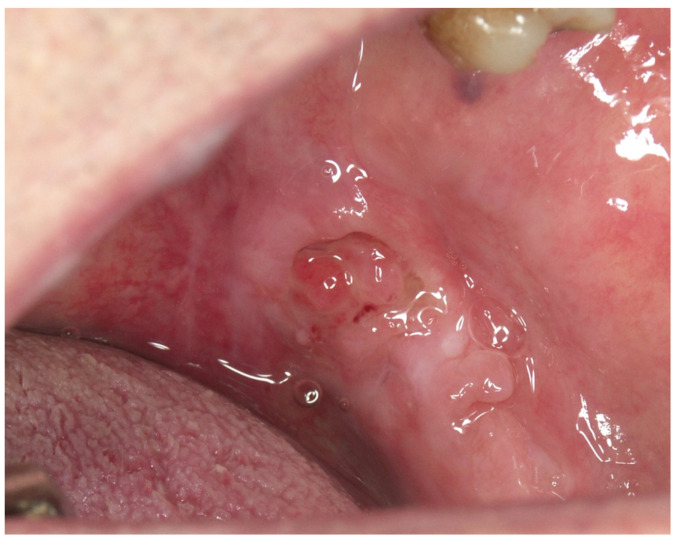
Ulcerated incipient oral carcinoma. Reproduced with permission from [67].

**Figure A17:**
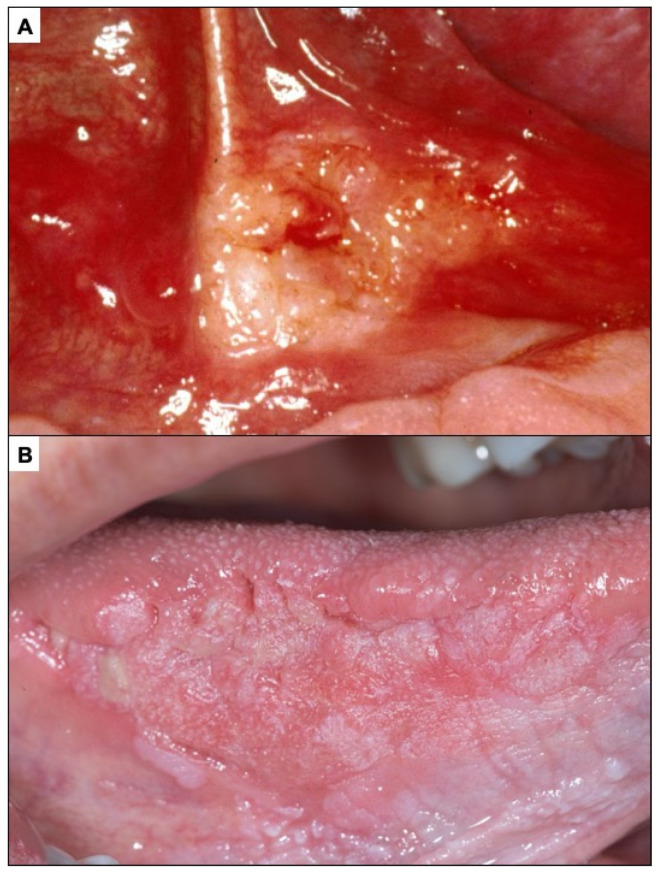
(**A**) Incipient oral carcinoma appearing as a raised lesion. (**B**) Oral carcinoma with granular aspect. Reproduced with permission from [67].

## Appendix A. Clinical Presentation of OPMD

Oral leukoplakia is the best-known OPMD. It is defined as a predominantly white plaque at risk for cancer development in which diagnosis is determined once other known diseases or disorders that do not carry a higher cancer risk than that existing in the general population have been excluded [53] (Figure A1). The most recent studies, with evidence-based designs [64], indicate that the malignancy rate of oral leukoplakias exceeds 9% of cases, indicating that a patient with this lesion has a significant risk of developing cancer; however, there is no evidence as to what proportion of oral carcinomas developed on leukoplakias are diagnosed late and what are the reasons for their late diagnosis. Presumably, carcinomas that evolve on leukoplakia may be diagnosed late for one of the following reasons.

A misdiagnosis of oral leukoplakia confused with other white lesions without the risk of cancer undoubtedly entails a risk of underestimating the relevance of the lesion and of not applying the appropriate procedures for its treatment and follow up. The most relevant lesions that could be confused with leukoplakia are outlined below.

Pseudomembranous candidiasis. It is a *Candida albicans* infection characterized by the presence of white aggregates with an appearance similar to milk or yogurt clots, which has the essential characteristic of detaching when the lesion is scraped with a gauze or spatula. Pseudomembranous candidiasis requires for its development a state of physical weakness or immunosuppression, which may be conditioned by the presence of some chronic diseases (for example, diabetes or immunodeficiencies), advanced age, or the intake of some drugs, among which include broad-spectrum antibiotics and topical or systemic immunosuppressants [65] (Figure A2).

Frictional lesions appear in the oral cavity as a consequence of the continuous friction of aggressive agents on the oral mucosa, the most common being destroyed teeth with sharp edges and prostheses in poor condition. Frequently, if it is a very aggressive agent (for example, a sharp tooth), it is common to observe an ulcerative lesion of benign characteristics (homogeneous background and well-defined edges surrounded by a white halo). It is also common to find frictional lesions on the alveolar ridges with the prolonged absence of teeth, which is due to the chronic trauma of mastication. In the clinical experience of the authors of this paper, these frictional lesions were frequently confused with leukoplakia, and incorrect and unnecessary procedures such as biopsy of the lesion or informing the patient about the premalignant nature of the lesion are performed, causing alarm and unnecessary economic expense. The diagnosis of a frictional lesion is purely clinical and requires identifying the causal agent, eliminating it, and checking the lesion, which should disappear in approximately ten days; if this does not happen, it is then imperative to biopsy the lesion to rule out an incipient carcinoma; we must not forget that some neoplasms at the beginning of their evolution could simulate frictional lesions (Figure A3 and Figure A4).

Leukoedema is a variant of normal characterized by the presence of bilateral opaque-lescent lesions that disappear when the jugal mucosa is tracted with examination mirrors. It is sometimes accompanied by a white line in the middle zone of the buccal mucosa (alba line). None of these variants of normality should be mistaken for leukoplakia and, consequently, should not be biopsied, referred to specialists, nor, of course, should we inform the patient about premalignancy (Figure A5 and Figure A6).

White lesions are associated with syndromes without premalignant character, i.e., white spongioid nevus, which is a white lesion that is inherited in an autosomal dominant manner. It is widely distributed in the oral mucosa, and its clinical characteristic is that it appears in childhood (Figure A7). The type of inheritance with which it is transmitted implies that some of the progenitors should also present similar lesions in the oral mucosa. The diagnosis is essentially clinical, although doubtful cases should be biopsied. There is no risk of malignancy, and no treatment is required, although, in the case of children, it is necessary that the parents are accurately informed about the process. The appearance of white lesions on the oral mucosa of children is always worrying. In newborns, they could be pseudomembranous candidiasis; in older children, a white lesion of the oral mucosa should always arouse the suspicion of a syndromic picture as a cause since oral leukoplakia in these ages is practically nonexistent. These syndromic pictures are not always as innocent as the white spongiotic nevus; thus, dyskeratosis congenita, which associates oral lesions of leukoplakic appearance in childhood or adolescence, nail dystrophy, and melanic pigmentations of reticulated appearance in the skin, has a premalignant character and can evolve to oral cancer in early life [66] (Figure A6 and Figure A8).

An oral carcinoma developed on a leukoplakia could be diagnosed late as a consequence of ignorance of the clinical facts indicating malignancy. Clinicians should be attentive to the appearance of indurated, raised, ulcerated, granular, or reddened [67] lesions during the evolution of a leukoplakia, detected during the obligatory follow up that these patients must undergo. In these cases, a biopsy is imperative (Figure A9).

The absence of a biopsy in an oral leukoplakia could delay the diagnosis of cancer; it should be kept in mind that approximately 12% of carcinomas present in their initial stages as leukoplakia [67]. On the other hand, a biopsy of a leukoplakia could result in the presence of severe dysplasia or carcinoma in situ, both histopathologic features that strongly predispose to the development of cancer [68] (Figure A10 and Figure A11).

The failure to follow patients with oral leukoplakia may delay the diagnosis of cancer. Patients with oral leukoplakia that has recurred after surgical treatment or those with lesions so extensive or multifocal that complete removal is impossible should probably be followed for life. Even patients who have been successfully treated should be followed up with for some time to ensure that the lesions do not recur as new leukoplakia or even as carcinomas. Failure to establish a follow-up program may be due to poor training of the clinician, who is unaware of this aspect to saturation, of the public health systems or to a lack of collaboration of the patient. It should also be taken into account that there is no evidence as to the best follow-up program applicable to these patients.

Oral lichen planus (OLP) is a very prevalent OPMD (1% of the general population) [69] with a malignancy rate of 1.14% of cases, which increases when erythematous or atrophic lesions develop, affecting the tongue, in patients who smoke or in those infected with hepatitis C virus [70,71,72]. OLP lesions present with a white striated appearance that may or may not be accompanied by erythematous or erosive lesions. Occasionally, OLP may also present with white leukoplakia-like plaques, with papular or even bullous lesions [73,74] (Figure A12). The clinical signs that should lead to suspecting the development of cancer on OLP are essentially the appearance of red areas, ulcers of neoplastic aspect, indurations of the tissue, or exophytic lesions (Figure A13). Among the reasons that could justify delaying the diagnosis of oral cancer that has developed on OLP include the following:

-There is non-consideration by some clinicians of OLP as an OPMD. This fact, largely overcome by recent evidence [70,71,72], is nevertheless still applied by some clinicians [75]. It is axiomatic that if a clinician does not consider a lesion as an OPMD, they will never be able to diagnose early its malignancy [67].

-On the other hand, the diagnostic criteria for OLP proposed by van de Waal’s group [76] that have been widely followed by many clinicians and researchers included the consideration of epithelial dysplasia as an exclusion criterion for OLP, and this, in our opinion, is not only unjustified, having been questioned by current evidence, but also implies a serious risk of underestimating the malignant potential of the disease [70,77], since it has been shown [70,78] that the presence of epithelial dysplasia is the fact that most powerfully affects the risk of OLP malignancy.

-In the same way, it is now accepted that the diagnosis of OLP requires the joint consideration of clinical findings (essentially the presence of white reticular lesions) together with histopathologic findings that, after biopsy of the diseased tissue, demonstrate aspects of an autoimmune phenomenon (inflammatory band infiltration and vacuolizing degeneration of the basal layer of the epithelium). Our group’s works [70] have shown that case series published with the aim of analyzing the malignancy rate of OLP report higher rates if they base the diagnosis of the disease on the conjunction of clinical and histopathological aspects, while those series that only base the diagnosis on clinical facts find significantly lower rates of malignancy. In our opinion, the reason for this result is due to the fact that biopsy can detect incipient carcinomas presenting as red areas that, if not biopsied, could easily be confused with the erythematous areas frequently present in OLP; likewise, a granular aspect of the mucosa, frequently present in early oral cancer, could also be confused with papular areas that occasionally appear in OLP. The conclusion of this observation is that OLP lesions should be biopsied in the diagnostic process, and the sampling should always include red areas.

-The lack of knowledge of the disease on the part of dentists, family physicians, or other health professionals, or the lack of knowledge of the premalignant nature of OLP, in the personal experience of the authors, is a common occurrence. Consequently, it is also common for patients not to be followed up adequately. Moreover, there is a lack of awareness on the part of dentists, family physicians, or other healthcare professionals of the clinical signs suggestive of cancer development on an OLP.

-Finally, hypothetically, the absence of adequate treatment of the disease based essentially on local immunosuppression could facilitate the malignant transformation of the lesions since it is assumed that the malignant process is very probably associated with immune aggression. However, there is not enough evidence of this.

Proliferative verrucous leukoplakia (PVL) is another relevant OPMD due to its high malignancy rate, estimated to be close to 50% of cases [79], and the possibility of multiple carcinomas developing in one patient [79,80]. PVL initially presents as localized white lesions which, over time and progressively, spread to large areas of the oral mucosa, eventually affecting multiple locations, almost always including the gingiva and palate, acquiring in some areas verrucous or even erythematous aspects (Figure A14). This disease most frequently affects women who do not present risk factors; that is, they are not smokers or drinkers [81]. The difficulty in the early diagnosis of oral cancer that develops from PVL is due to its initial innocuous appearance, which could make it appear as an innocent lesion; its diagnosis made retrospectively after a long period of evolution, which could relax the principles of follow-up in the initial stages of the disease; the large extension of the lesions when they are found in the early stages of the disease; it not being present in the early stages of the disease; and it not being diagnosed in the early stages of the disease. The large extent of the lesions when the disease is fully established, which probably makes clinical examination and the detection of any worrisome clinical aspects that may appear difficult, as well as the very prolonged evolution of the lesions and their frequent recurrence after treatment, which could discourage patients from complying with follow-up programs and even discourage clinicians themselves from applying them. The lack of knowledge of this lesion on the part of dentists, family physicians, otorhinolaryngologists, and other specialists in the area must undoubtedly also justify the late diagnosis of the malignization of this disease.

Erythroplasia is defined as an intensely red plaque without specific features that allow it to be classified as other red lesions without risk of progression to cancer [53]. Diagnosing erythroplasia is a very serious clinical event since we have known for some time that approximately 70% of them are established carcinomas, carcinomas in situ, or have severe dysplasia on the histologic study [53]. The differential diagnosis of oral erythroplasia is complex because numerous inflammatory and traumatic lesions of the oral mucosa can manifest with an erythematous appearance. The presence of well-defined boundaries in a lesion involving a localized area of the oral mucosa suggests erythroplasia, and, in this sense, the observation of a red area of the oral mucosa should always constitute a cause for alarm. The reason for a late diagnosis of oral cancer in this lesion lies in the rarity of erythroplasia, which consequently leads to a remarkable lack of knowledge of the disease. Moreover, the fact that they often harbor severe dysplasia or carcinoma in situ favors their rapid progression to invasive carcinoma.

Our research group, in two evidence-based studies [80,82], has shown that carcinomas on OLP and PVL behave better compared to conventional oral carcinomas. The reason for this is unknown, although it is probably due to reasons related to the biopathology of the tumors they develop, which are generally better differentiated. It is improbable and debatable that the better prognosis is due to the close follow up of these patients, which has not been demonstrated in the published series [80,82].

Oral carcinomas are initially present as localized red areas in more than 60% of cases (Figure A15A). About 12% of cases have leukoplakia-like lesions (Figure A15B) and may also appear as a mixture of red and white lesions (Figure A15C). The presence of ulcerations of more than one month of evolution, non-traumatic, should suggest cancer (Figure A16), especially if they are indurated, with irregular raised borders and irregular and distorted floors. It is possible for an incipient oral carcinoma to also present as a raised lesion (Figure A17A) or a granular lesion (Figure A17B). It is imperative that clinicians become familiar with these lesions, train themselves in the knowledge of their clinical appearance by visiting pathology atlases, and, above all, always think that an unexplained lesion of the oral mucosa could be cancer, thus avoiding complacency and showing a diligent and proactive attitude in the early diagnosis of oral cancer.

### General Principles of Treatment of the Main OPMDs

The treatment of the main OPMD involves eliminating the etiological factors related to their development. This is feasible essentially with regard to oral leukoplakia, which we now know to be closely related to tobacco consumption; thus, in those cases of leukoplakia in which smoking is identified as an etiological factor, it should be eliminated. Smoking cessation causes the disappearance of 70% of leukoplakia in the course and, therefore, also progressively decreases the risk of malignant transformation of these lesions [83,84]. The beneficial effect of this measure on the prognosis of tobacco-associated leukoplakias justifies explicitly informing patients of the importance of this aspect. However, we also know that smoking cessation is not easy for most patients, and it is sometimes necessary for them to undergo adjuvant pharmacological treatment or psychological support therapies. Leukoplakias are not associated with smoking, and those that do not disappear after smoking cessation should be removed, although in some cases, this is notoriously difficult or impossible due to the location or extent of the lesions. Some leukoplakias recur after surgical removal, and there is still insufficient evidence on how to proceed in these cases. Finally, patients with oral leukoplakia should be evaluated periodically for an indefinite period of time for which there is not enough evidence available, in anticipation of the recurrence of the lesion in the initial location or in other intraoral locations, and to detect early the development of cancer in any part of the oral mucosa, which risk is increased in these patients [83,84].

The treatment of erythroplasia is an urgent matter in oral medicine due to the high proportion of cases presenting severe dysplasia, carcinoma in situ, or frankly invasive carcinoma on histological examination. It should be remembered that the red lesion is the most frequent clinical sign of early oral carcinoma. In erythroplasia, the treatment of choice is the complete surgical removal of the lesion, patient follow up, and cessation of tobacco consumption, which is again the main risk factor for its development [85].

Oral lichen planus is, together with oral leukoplakia, one of the most frequent potentially malignant oral disorders encountered in clinical practice [69]. We know that approximately 50% of the cases are asymptomatic, pure reticular lesions, which do not present a risk of progression to cancer [69]. Therefore, these lesions do not require treatment, although they do require follow-up for an indefinite period of time because we have no evidence that they remain as pure reticular lesions throughout the patient’s life [70]. Atrophic and erosive oral lichen planus is usually symptomatic, presents a risk of evolution to cancer, and, for this reason, should be treated. Due to its autoimmune nature, the treatment of choice is the application of topical corticotherapy. We do not have sufficient evidence on the best regimen or the most appropriate topical corticosteroid to control the disease, although the available data point to clobetasol propionate as the most effective corticosteroid. Patients with oral lichen planus should be followed for life in an attempt to diagnose early the development of cancer on these lesions, although as in other potentially malignant oral disorders, there is not enough evidence on the most appropriate follow-up regimen [86].
